# Crystal structure of (1*E*,1′*E*)-1,1′-(pyridine-2,6-di­yl)bis­[*N*-(2,3,4,5,6-penta­fluoro­phen­yl)ethan-1-imine]

**DOI:** 10.1107/S2056989017008040

**Published:** 2017-06-02

**Authors:** Jenna Boyle, Catherine Breakfield, Leah Buck, Catherine McMahon, Dominic C. Babbini

**Affiliations:** a Department of Chemistry and Physics, Saint Mary’s College, Notre Dame, IN 46556, USA

**Keywords:** crystal structure, pyridine di­imine, redox non-innocent ligand, electron-withdrawing groups, Schiff base

## Abstract

The title compound, C_21_H_9_F_10_N_3_, represents a potential redox non-innocent pyridine di­imine ligand system. It consists of a central pyridine ring with two penta­fluoro­phenyl substituted imine groups in positions 2 and 6. The whole mol­ecule is generated by mirror symmetry, the mirror bis­ecting the N and *para*-C atom of the pyridine ring. The perfluoro­phenyl ring is inclined to the pyridine ring by 73.67 (8)°. In the crystal, mol­ecules stack along the *a* axis, but there are no significant inter­molecular inter­actions present.

## Abstract 2

The synthesis and crystal structure of a potentially redox non-innocent penta­fluoro­phenyl-substituted pyridine di­imine ligand system are reported.

## Chemical context   

The utilization of non-innocent ligand systems in organometallic chemistry can produce secondary reactivity and can result in unique mechanistic and redox properties (Babbini & Iluc, 2015 ▸; Praneeth *et al.*, 2012 ▸). Redox non-innocence is generally observed with chelate ligands which possess low-lying π-systems that can allow electron transfer (Lyaskovskyy & de Bruin, 2012 ▸). These ligand systems allow multiple-electron redox events to take place on metal centers, which are usually relegated to single-electron events (Haneline & Heyduk, 2006 ▸). This is useful for the utilization of benign and economically viable first-row transition metals instead of traditional noble-metal catalysts (Chirik & Wieghardt, 2010 ▸). The development of new and varied ligands systems is essential for the understanding of the structure–property relationships, which give rise to redox non-innocence. Given the significance and current inter­est in redox-active ligand systems, herein we report on the synthesis and crystal structure of a potential redox-active pyridine di­imine system containing electron-withdrawing substituents.
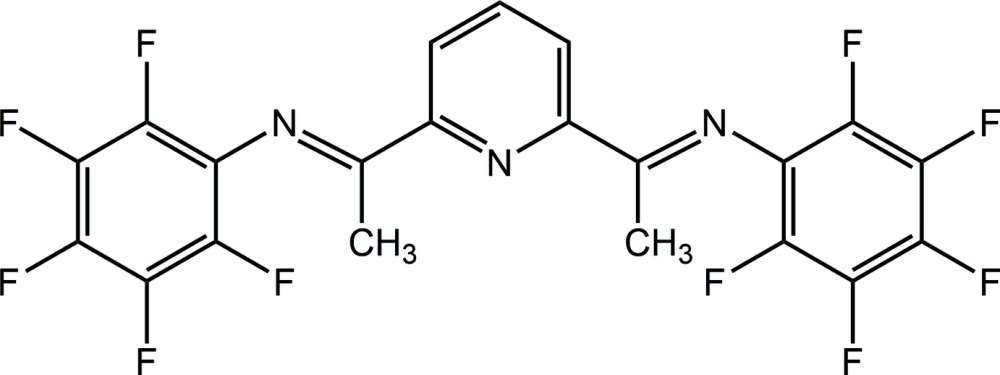


## Structural commentary   

**Figure 1:**
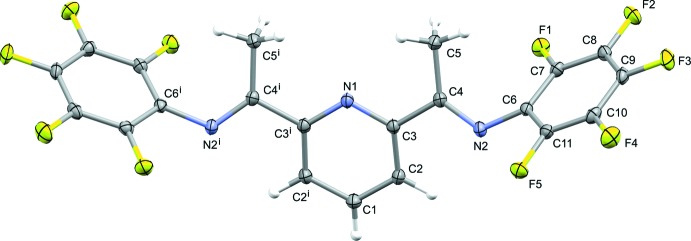
The mol­ecular structure of the title compound, with the atom labelling [symmetry code: (i) *x*, −*y* + 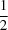, *z*]. Displacement ellipsoids are drawn at the 50% probability level.

The title compound, Fig. 1 ▸, crystallizes in the monoclinic space group *P*2_1_/*m* with the mirror plane, at (*x*, 0.25, *z*), bis­ecting the pyridine N atom, N1, and C atom, C1. Thus, only half of the mol­ecule is present in the asymmetric unit (Fig. 1 ▸). The penta­fluoro­phenyl groups are oriented in a *synclinal* fashion with respect to the pyridine ring, with the two rings being inclined to one another by 73.67 (6)°. The imine nitro­gen atom, N2, is oriented in an *anti*-conformation with respect to the pyridine nitro­gen, N1. This orientation is in contrast with the mol­ecule acting as a tridentate ligand coordinating to the chromium ion in complex tri­chloro­(2,6-bis­(1-(penta­fluoro­phenyl­imino)­eth­yl)pyridine-*N*,*N*′,*N*′′)chromium(III) aceto­nitrile monosolvate (Nakayama *et al.*, 2005 ▸). Here, the imine N atoms adopt a *syn*-conformation upon coordination to the chromium ion.

## Supra­molecular features   

**Figure 2:**
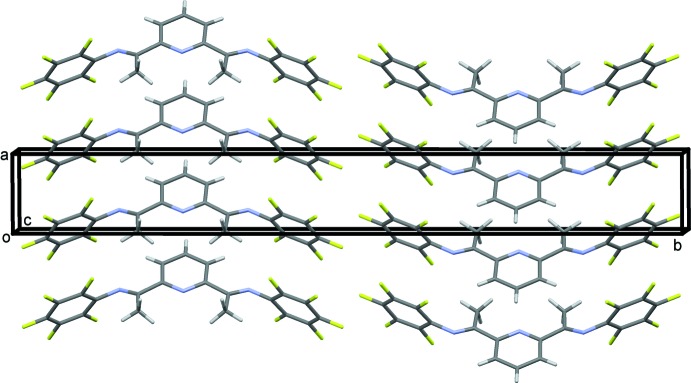
A view along the *c* axis of the crystal packing of the title compound.

In the crystal, the mol­ecules stack along the *a* axis (Fig. 2 ▸). Despite the presence of multiple aromatic rings within the mol­ecule, there are no obvious π-stacking inter­actions; the phenyl rings are clearly offset. Thus the only inter­molecular inter­actions present are typical van der Waals inter­actions.

## Database survey   

A search of the Cambridge Structural Database (CSD, V5.38, last update February 2017; Groom *et al.*, 2016 ▸) for related structures reveals that the penta­fluoro­phenyl adduct reported here has been reported as a chelating ligand in the chromium complex, tri­chloro­(2,6-bis­(1-(penta­fluoro­phenyl­imino)­eth­yl)pyridine-*N*,*N*′,*N*′′)chromium(III) aceto­nitrile monosolvate (CSD refcode: BOMROL; Nakayama *et al.*, 2005 ▸). The mesityl and 2,6-diiso­propyl­phenyl species are well represented and the solid-state structures of these free mol­ecules have been reported; *viz.* SISYEA (Boyt & Chaplin, 2014 ▸) and HORSEM (Yap & Gambarotta, 1999 ▸), respectively.

## Synthesis and crystallization   

The reagent 2,6-di­acetyl­pyridine was synthesized by a previously reported method (Su & Feng, 2010 ▸), and the ligand was prepared by a modification of a previously reported Schiff-base condensation method (Small & Brookhart, 1999 ▸).

A mixture of 2,6-di­acetyl­pyridine (1.0 g, 6.10 mmol), 2,3,4,5,6-penta­fluoro­aniline (4.07 g, 22.2 mmol) and *p*-tol­uene­sulfonic acid (10 mg, 0.058 mmol) in toluene (100 ml) was refluxed for 30 h during which time water was removed by a Dean–Stark apparatus. The crude yellow product was washed with cold methanol and filtered producing a pure off-white solid (yield 1.65 g, 54.8%). Colorless block-like crystals were obtained by vapor diffusion of hexa­nes into a saturated di­chloro­methane solution of the title compound. Spectroscopic data: ^1^H NMR (60 MHz, CDCl_3_): δ 8.6–7.8 (*m*, 3H, Py-*H*), 2.5 (*s*, 6H, C*H*_3_), and MS (ESI): *m*/*z* 494 [C_21_H_9_F_10_N_3_]H^+^.

## Refinement   

Crystal data, data collection and structure refinement details are summarized in Table 1 ▸. The hydrogen atoms were included in calculated positions and refined with a riding model: C—H = 0.95–0.98 Å with *U*_iso_(H) = 1.5*U*_eq_(C-meth­yl) and 1.2*U*_eq_(C) for other H atoms.

## Supplementary Material

Crystal structure: contains datablock(s) I. DOI: 10.1107/S2056989017008040/su5374sup1.cif

Structure factors: contains datablock(s) I. DOI: 10.1107/S2056989017008040/su5374Isup2.hkl

Supporting information file. DOI: 10.1107/S2056989017008040/su5374Isup3.cml

CCDC reference: 1553189

Additional supporting information:  crystallographic information; 3D view; checkCIF report

## Figures and Tables

**Table 1:** Experimental details

Crystal data	
Chemical formula	C_21_H_9_F_10_N_3_
*M*_r_	493.31
Crystal system, space group	Monoclinic, *P*2_1_/*m*
Temperature (K)	120
*a*, *b*, *c* (Å)	4.2713 (6), 35.792 (5), 5.9516 (9)
β (°)	93.326 (2)
*V* (Å^3^)	908.3 (2)
*Z*	2
Radiation type	Mo *K*α
μ (mm^−1^)	0.18
Crystal size (mm)	0.24 × 0.19 × 0.14

Data collection	
Diffractometer	Bruker APEXII CCD
Absorption correction	Multi-scan (*SADABS*; Krause *et al.*, 2015 ▸)
*T*_min_, *T*_max_	0.697, 0.729
No. of measured, independent and observed [*I* > 2σ(*I*)] reflections	13884, 2277, 1989
*R*_int_	0.025
(sin θ/λ)_max_ (Å^−1^)	0.666

Refinement	
*R*[*F* ^2^ > 2σ(*F* ^2^)], *wR*(*F* ^2^), *S*	0.034, 0.088, 1.07
No. of reflections	2277
No. of parameters	158
H-atom treatment	H-atom parameters constrained
Δρ_max_, Δρ_min_ (e Å^−3^)	0.36, −0.19

Computer programs: *APEX3* and *SAINT* (Bruker, 2016 ▸), *SHELXS97* (Sheldrick, 2008 ▸), *Mercury* (Macrae *et al.*, 2008 ▸), *SHELXL2016* (Sheldrick, 2015 ▸) and *publCIF* (Westrip, 2010 ▸).

## supplementary crystallographic information

### Crystal data

**Table:**

C_21_H_9_F_10_N_3_	*F*(000) = 492
*M**_r_* = 493.31	*D*_x_ = 1.804 Mg m^−^^3^
Monoclinic, *P*2_1_/*m*	Mo *K*α radiation, λ = 0.71073 Å
*a* = 4.2713 (6) Å	Cell parameters from 5126 reflections
*b* = 35.792 (5) Å	θ = 4.6–56.5°
*c* = 5.9516 (9) Å	µ = 0.18 mm^−^^1^
β = 93.326 (2)°	*T* = 120 K
*V* = 908.3 (2) Å^3^	Block, colorless
*Z* = 2	0.24 × 0.19 × 0.14 mm

### Data collection

**Table:**

Bruker APEXII CCD diffractometer	2277 independent reflections
Radiation source: sealed tube	1989 reflections with *I* > 2σ(*I*)
Graphite monochromator	*R*_int_ = 0.025
Detector resolution: 8.33 pixels mm^-1^	θ_max_ = 28.3°, θ_min_ = 2.3°
φ and ω scans	*h* = −5→5
Absorption correction: multi-scan (SADABS; Krause *et al.*, 2015)	*k* = −47→44
*T*_min_ = 0.697, *T*_max_ = 0.729	*l* = −7→7
13884 measured reflections	

### Refinement

**Table:**

Refinement on *F*^2^	Primary atom site location: structure-invariant direct methods
Least-squares matrix: full	Secondary atom site location: difference Fourier map
*R*[*F*^2^ > 2σ(*F*^2^)] = 0.034	Hydrogen site location: inferred from neighbouring sites
*wR*(*F*^2^) = 0.088	H-atom parameters constrained
*S* = 1.07	*w* = 1/[σ^2^(*F*_o_^2^) + (0.0377*P*)^2^ + 0.4768*P*] where *P* = (*F*_o_^2^ + 2*F*_c_^2^)/3
2277 reflections	(Δ/σ)_max_ = 0.001
158 parameters	Δρ_max_ = 0.36 e Å^−^^3^
0 restraints	Δρ_min_ = −0.19 e Å^−^^3^

### Special details

**Table:**

Experimental. All other reagents and solvents were purchased commercially and used without further purification. ^1^H NMR was collected on a Varian 60 MHz NMR. Mass spectra were collected using direct injection on a ThermoScientific TSQ-ESI Mass spectrometer.
Geometry. All esds (except the esd in the dihedral angle between two l.s. planes) are estimated using the full covariance matrix. The cell esds are taken into account individually in the estimation of esds in distances, angles and torsion angles; correlations between esds in cell parameters are only used when they are defined by crystal symmetry. An approximate (isotropic) treatment of cell esds is used for estimating esds involving l.s. planes.

### Fractional atomic coordinates and isotropic or equivalent isotropic displacement parameters (Å^2^)

**Table:**

	*x*	*y*	*z*	*U*_iso_*/*U*_eq_	
F1	0.42432 (19)	0.38311 (2)	0.12835 (13)	0.02066 (19)	
F2	0.2099 (2)	0.45211 (2)	0.02453 (13)	0.0251 (2)	
F3	−0.1849 (2)	0.48751 (2)	0.29875 (14)	0.0261 (2)	
F4	−0.3500 (2)	0.45363 (2)	0.68211 (14)	0.0254 (2)	
F5	−0.13051 (19)	0.38487 (2)	0.79035 (13)	0.02134 (19)	
N1	0.2566 (4)	0.250000	0.4081 (2)	0.0138 (3)	
N2	0.2674 (3)	0.34658 (3)	0.52933 (17)	0.0155 (2)	
C1	0.6985 (4)	0.250000	0.7721 (3)	0.0178 (4)	
H1	0.855045	0.250001	0.892210	0.021*	
C2	0.5833 (3)	0.28342 (3)	0.6836 (2)	0.0160 (3)	
H2	0.653646	0.306655	0.745044	0.019*	
C3	0.3612 (3)	0.28213 (3)	0.5019 (2)	0.0139 (2)	
C4	0.2187 (3)	0.31743 (3)	0.4075 (2)	0.0138 (2)	
C5	0.0259 (3)	0.31505 (4)	0.1893 (2)	0.0179 (3)	
H5A	−0.093139	0.338255	0.165094	0.027*	
H5B	−0.119674	0.293945	0.194596	0.027*	
H5C	0.164407	0.311399	0.065501	0.027*	
C6	0.1457 (3)	0.38157 (3)	0.4594 (2)	0.0137 (2)	
C7	0.2293 (3)	0.39993 (3)	0.2659 (2)	0.0149 (2)	
C8	0.1229 (3)	0.43548 (4)	0.2117 (2)	0.0165 (3)	
C9	−0.0740 (3)	0.45357 (3)	0.3516 (2)	0.0175 (3)	
C10	−0.1585 (3)	0.43632 (4)	0.5469 (2)	0.0170 (3)	
C11	−0.0465 (3)	0.40098 (3)	0.5999 (2)	0.0150 (2)	

### Atomic displacement parameters (Å^2^)

**Table:**

	*U*^11^	*U*^22^	*U*^33^	*U*^12^	*U*^13^	*U*^23^
F1	0.0216 (4)	0.0202 (4)	0.0210 (4)	0.0031 (3)	0.0075 (3)	−0.0019 (3)
F2	0.0339 (5)	0.0204 (4)	0.0213 (4)	−0.0026 (3)	0.0040 (3)	0.0081 (3)
F3	0.0326 (5)	0.0118 (4)	0.0330 (5)	0.0066 (3)	−0.0043 (4)	0.0023 (3)
F4	0.0243 (4)	0.0242 (4)	0.0284 (4)	0.0077 (3)	0.0070 (3)	−0.0070 (3)
F5	0.0270 (4)	0.0210 (4)	0.0164 (4)	−0.0022 (3)	0.0053 (3)	0.0018 (3)
N1	0.0163 (7)	0.0099 (7)	0.0152 (7)	0.000	0.0013 (5)	0.000
N2	0.0183 (5)	0.0106 (5)	0.0173 (5)	0.0005 (4)	−0.0009 (4)	0.0007 (4)
C1	0.0199 (9)	0.0156 (8)	0.0174 (8)	0.000	−0.0037 (7)	0.000
C2	0.0193 (6)	0.0112 (6)	0.0172 (6)	−0.0009 (4)	−0.0007 (5)	−0.0010 (4)
C3	0.0155 (6)	0.0116 (6)	0.0146 (5)	−0.0004 (4)	0.0019 (4)	0.0005 (4)
C4	0.0145 (6)	0.0120 (5)	0.0150 (5)	−0.0003 (4)	0.0011 (4)	0.0008 (4)
C5	0.0225 (7)	0.0134 (6)	0.0172 (6)	0.0009 (5)	−0.0040 (5)	−0.0010 (4)
C6	0.0145 (6)	0.0105 (5)	0.0156 (6)	−0.0001 (4)	−0.0026 (4)	−0.0009 (4)
C7	0.0146 (6)	0.0138 (6)	0.0163 (6)	0.0003 (4)	0.0013 (4)	−0.0027 (4)
C8	0.0182 (6)	0.0146 (6)	0.0166 (6)	−0.0026 (5)	−0.0004 (5)	0.0031 (5)
C9	0.0188 (6)	0.0097 (5)	0.0234 (6)	0.0014 (5)	−0.0045 (5)	−0.0003 (5)
C10	0.0153 (6)	0.0160 (6)	0.0196 (6)	0.0020 (5)	0.0008 (5)	−0.0053 (5)
C11	0.0164 (6)	0.0146 (6)	0.0137 (5)	−0.0034 (5)	−0.0001 (4)	−0.0003 (4)

### Geometric parameters (Å, º)

**Table:**

F1—C7	1.3439 (14)	C2—H2	0.9500
F2—C8	1.3348 (15)	C3—C4	1.4974 (16)
F3—C9	1.3347 (14)	C4—C5	1.4995 (17)
F4—C10	1.3330 (15)	C5—H5A	0.9800
F5—C11	1.3391 (14)	C5—H5B	0.9800
N1—C3^i^	1.3432 (14)	C5—H5C	0.9800
N1—C3	1.3432 (14)	C6—C7	1.3902 (17)
N2—C4	1.2806 (16)	C6—C11	1.3913 (17)
N2—C6	1.4098 (15)	C7—C8	1.3829 (18)
C1—C2	1.3857 (15)	C8—C9	1.3787 (19)
C1—C2^i^	1.3858 (15)	C9—C10	1.3822 (19)
C1—H1	0.9500	C10—C11	1.3824 (18)
C2—C3	1.3976 (17)		
			
C3^i^—N1—C3	117.76 (15)	H5B—C5—H5C	109.5
C4—N2—C6	120.78 (10)	C7—C6—C11	116.79 (11)
C2—C1—C2^i^	119.37 (16)	C7—C6—N2	123.82 (11)
C2—C1—H1	120.3	C11—C6—N2	119.10 (11)
C2^i^—C1—H1	120.3	F1—C7—C8	118.50 (11)
C1—C2—C3	118.40 (12)	F1—C7—C6	119.40 (11)
C1—C2—H2	120.8	C8—C7—C6	122.09 (12)
C3—C2—H2	120.8	F2—C8—C9	120.24 (11)
N1—C3—C2	122.98 (11)	F2—C8—C7	120.09 (12)
N1—C3—C4	116.64 (11)	C9—C8—C7	119.67 (12)
C2—C3—C4	120.35 (11)	F3—C9—C8	120.38 (12)
N2—C4—C3	115.20 (11)	F3—C9—C10	119.88 (12)
N2—C4—C5	126.84 (11)	C8—C9—C10	119.74 (11)
C3—C4—C5	117.90 (10)	F4—C10—C9	119.96 (11)
C4—C5—H5A	109.5	F4—C10—C11	120.23 (12)
C4—C5—H5B	109.5	C9—C10—C11	119.80 (12)
H5A—C5—H5B	109.5	F5—C11—C10	118.76 (11)
C4—C5—H5C	109.5	F5—C11—C6	119.36 (11)
H5A—C5—H5C	109.5	C10—C11—C6	121.87 (12)
			
C2^i^—C1—C2—C3	−2.2 (3)	F1—C7—C8—C9	−179.14 (11)
C3^i^—N1—C3—C2	3.3 (2)	C6—C7—C8—C9	−0.40 (19)
C3^i^—N1—C3—C4	−174.53 (9)	F2—C8—C9—F3	1.46 (19)
C1—C2—C3—N1	−0.6 (2)	C7—C8—C9—F3	−178.34 (11)
C1—C2—C3—C4	177.14 (13)	F2—C8—C9—C10	−178.96 (11)
C6—N2—C4—C3	−179.98 (11)	C7—C8—C9—C10	1.24 (19)
C6—N2—C4—C5	−2.7 (2)	F3—C9—C10—F4	−0.11 (18)
N1—C3—C4—N2	164.92 (13)	C8—C9—C10—F4	−179.69 (11)
C2—C3—C4—N2	−12.97 (18)	F3—C9—C10—C11	179.21 (11)
N1—C3—C4—C5	−12.59 (17)	C8—C9—C10—C11	−0.38 (19)
C2—C3—C4—C5	169.52 (12)	F4—C10—C11—F5	−0.92 (18)
C4—N2—C6—C7	−62.67 (17)	C9—C10—C11—F5	179.76 (11)
C4—N2—C6—C11	123.67 (13)	F4—C10—C11—C6	177.94 (11)
C11—C6—C7—F1	177.46 (10)	C9—C10—C11—C6	−1.38 (19)
N2—C6—C7—F1	3.67 (18)	C7—C6—C11—F5	−178.99 (11)
C11—C6—C7—C8	−1.27 (18)	N2—C6—C11—F5	−4.89 (17)
N2—C6—C7—C8	−175.06 (11)	C7—C6—C11—C10	2.15 (18)
F1—C7—C8—F2	1.06 (18)	N2—C6—C11—C10	176.25 (11)
C6—C7—C8—F2	179.80 (11)		

Symmetry code: (i) *x*, −*y*+1/2, *z*.
